# Correlation of Cystatin C- and Creatinine-Based Renal Equations With Lipid Parameters in Chronic Kidney Disease: A Case-Control Study

**DOI:** 10.7759/cureus.113123

**Published:** 2026-07-21

**Authors:** Smita H Vasava, Roshni G Sadaria, Devanshi P Gosai

**Affiliations:** 1 Department of Biochemistry, Pramukhswami Medical College, Bhaikaka University, Anand, IND; 2 Department of Biochemistry, Smt. B. K. Shah Medical Institute and Research Centre, Sumandeep Vidyapeeth Deemed to be University, Vadodara, IND; 3 Department of Pathology, Smt. B. K. Shah Medical Institute and Research Centre, Sumandeep Vidyapeeth Deemed to be University, Vadodara, IND

**Keywords:** cardiovascular risk, chronic kidney disease, cystatin c, estimated glomerular filtration rate, lipid profile

## Abstract

Introduction

Dyslipidemia is a significant risk factor for cardiovascular morbidity and mortality in patients with chronic kidney disease (CKD). Therefore, it is essential to identify cardiovascular risk at the earliest. Cystatin C is a novel biomarker due to its high sensitivity relative to creatinine for estimating glomerular filtration rate (GFR).

Aim

This study aimed to assess the correlation between cystatin C- and creatinine-based eGFR (using the Chronic Kidney Disease Epidemiology Collaboration (CKD-EPI) and Modification of Diet in Renal Disease (MDRD) equations) and lipid parameters (total cholesterol (TC), triglyceride (TG), low-density lipoprotein cholesterol (LDL-C), high-density lipoprotein cholesterol (HDL-C), very-low-density lipoprotein cholesterol (VLDL-C)) in adult CKD patients versus age- and sex-matched healthy controls.

Materials and methods

This case-control study was carried out at Dhiraj General Hospital, Piparia, Gujarat, India. A total of 100 participants were enrolled, including 50 CKD patients and 50 healthy controls. Serum creatinine, cystatin C, and lipid profile were measured. eGFR was calculated using MDRD (creatinine-based) and CKD-EPI (cystatin C-based) equations. Correlations between creatinine- and cystatin C-based eGFR with lipid parameters were assessed.

Results

CKD patients demonstrated significantly higher serum creatinine (11.64±5.86 mg/dl) and cystatin C (6.39±2.11 mg/dl) levels compared to controls. Patients with CKD had significantly elevated levels of serum TG and VLDL-C, whereas HDL-C was significantly lower compared to the controls (p<0.05). TC, LDL-C, TC/HDL ratio, and LDL/HDL ratio showed no statistically significant differences in the study groups (p>0.05). Correlation analysis revealed no significant association between cystatin C-based and creatinine-based eGFR with lipid parameters (p>0.05). However, cystatin C-based eGFR showed relatively stronger negative correlation with TC (r=-0.1603), LDL-C (r=-0.1913), and LDL/HDL ratio (r=-0.1855) compared with creatinine-based eGFR.

Conclusion

Correlations between eGFR and lipid parameters were weak and not statistically significant; however, cystatin C-based eGFR showed a relatively stronger, though non-significant, association with lipid parameters compared with creatinine-based eGFR. Larger studies are required to further explore these relationships.

## Introduction

In the 21st century, chronic kidney disease (CKD) has emerged as a major global health challenge and a significant contributor to mortality and morbidity worldwide. According to the World Health Organization (WHO), CKD will become the fifth most common chronic disorder by 2040. Diabetes mellitus, hypertension, coronary artery disease, and heart failure are the most frequent complications that occur in CKD [1]. CKD is diagnosed by glomerular filtration rate (GFR) <60 mL/min/1.73 m^2^ and/or the presence of proteinuria (urine albumin-to-creatinine ratio >30 mg/gm) and a health condition present for more than three months [2].

Serum creatinine is commonly used by consultants to monitor renal disease progression and treatment response due to its cost-effectiveness. Compared to creatinine, cystatin C is a novel biomarker and is considered superior to serum creatinine in the estimation of renal function as it is not influenced by age, sex, and body mass. A low-molecular-weight (13 kDa) cysteine proteinase inhibitor is produced at a constant rate by all nucleated cells. It is freely filtered by glomeruli and is almost completely reabsorbed and catabolized in the proximal tubules, with negligible urinary excretion [3].

The gold standard method for the estimation of GFR is an invasive exogenous marker such as iohexol or iothalamate. But clinically it is not widely used. Most commonly, creatinine-based estimated glomerular filtration rate (eGFR) is widely used. Creatinine is affected by multiple factors such as age, sex, and muscle mass. Cystatin C is a reliable alternative to creatinine for assessing renal function, as it is less influenced by muscle mass and a more accurate marker for GFR estimation [4].

Dyslipidemia in CKD patients often progresses with altered renal function and the advancement of cardiovascular risk. Recent studies find that lipid derangement was positively correlated with the severity of the disease and also advanced stages of CKD [5]. Studies have demonstrated that cystatin C-based eGFR is more strongly associated with cardiovascular events and mortality than creatinine-based eGFR [4]. As CKD advances toward end-stage renal disease, it imposes a significant burden of cardiovascular mortality and morbidity. As a result, patients with CKD are often more likely to die due to cardiovascular complications before reaching end-stage renal disease [6]. Hence, timely diagnosis of dyslipidemia is essential for improving patient outcomes.

Multiple studies have been done to see the correlation between cystatin C and creatinine. But links between eGFR using cystatin C and cardiovascular risk in CKD were very rarely studied. The present study aimed to assess the correlation between cystatin C- and creatinine-based eGFR (using the Chronic Kidney Disease Epidemiology Collaboration (CKD-EPI) and Modification of Diet in Renal Disease (MDRD) equations, respectively) and lipid parameters (total cholesterol (TC), triglyceride (TG), low-density lipoprotein cholesterol (LDL-C), high-density lipoprotein cholesterol (HDL-C), and very-low-density lipoprotein cholesterol (VLDL-C)) in adult patients with CKD compared to healthy age- and sex-matched controls. The findings of the study give us insights into whether cystatin C- or creatinine-based eGFR is a more reliable marker for the diagnosis of CKD and a better predictor of cardiovascular risk.

## Materials and methods

The present case-control study was carried out at Dhiraj General Hospital, Piparia, Gujarat, India, from April 2020 to March 2021. Ethical approval was granted by Sumandeep Vidyapeeth Institutional Ethics Committee (SVIEC) (approval number: SVIEC/ON/MEDI/RP/20018; date: April 28, 2020). A total of 100 subjects were enrolled, of which 50 patients with CKD were included as cases and 50 age- and sex-matched healthy individuals were included as controls.

Inclusion and exclusion criteria

Patients who visited the medicine inpatient or outpatient departments were included in the study. Also included were CKD patients aged 18-70 years and willing to participate in the study. In contrast, those with acute renal failure, thyroid disorders, steroid therapy, and malignancy, aged under 18 or over 70 years, and who were pregnant and unwilling to participate were excluded from the study. Informed written consent was obtained from each participant, and those willing to participate were enrolled in the study. CKD was diagnosed by clinicians according to the National Kidney Foundation's Kidney Disease Outcomes Quality Initiative guidelines.

The study sample size was determined by considering the feasibility and availability of CKD patients. Participants were enrolled by consecutive sampling. In total, 50 CKD cases and 50 healthy controls were included in the study.

Study procedure

Approximately 5 mL of fasting blood sample in a plain vacutainer was collected from the participants under aseptic precautions. Blood samples were allowed to clot for 30 minutes and centrifuged at 3000 rpm for 15 minutes. Serum was analyzed for renal function and lipid profile, including serum creatinine and cystatin C, TC, TG, and HDL-C. Serum cystatin C was determined using a Mispa-i2 analyzer (Agappe Diagnostics, Kochi, Kerala, India), and serum creatinine was determined using an EM 200 fully automated chemistry analyzer (Erba Mannheim, Mannheim, Germany). Serum cystatin C and creatinine levels were assessed using nephelometric and enzymatic methods, respectively. TC, TG, and HDL-C were analyzed by cholesterol oxidase peroxidase (CHOD-POD), glycerol phosphate oxidase (GPO), and polyethylene glycol methyl ether (PEGME) methods, respectively. Lipid parameters were analyzed on an EM 200 fully automated analyzer. LDL-C was calculated using Friedewald's formula. TC/HDL ratio and LDL/HDL ratio were also calculated.

Calculations

eGFR was calculated using the MDRD formula [3]: $\text{eGFR (males)}=175\times\text{standard SCr}^{-1.154}\times\text{(age in years)}^{-0.203}$ (if male) and $\text{eGFR (females)}=175\times\text{standard SCr}^{-1.154}\times\text{(age in years)}^{-0.203}\times0.742$ (if female).

eGFR was calculated using the CKD-EPI formula [3]: $\text{eGFR (males)}=127.7\times\mathrm{Scys}^{-1.17}\times\text{(age in years)}^{-0.13}$ (if male) and $\text{eGFR (females)}=127.7\times\mathrm{Scys}^{-1.17}\times\text{(age in years)}^{-0.13}\times0.91$ (if female).

Here, SCr means serum creatinine and Scys means serum cystatin-C.

Statistical analysis

The data were analyzed using IBM SPSS Statistics for Windows, Version 30.0 (IBM Corp., Armonk, New York, United States). Quantitative data were presented as mean±standard deviation (SD). Comparison and correlation between the two groups were evaluated using Student's t-test. Differences between independent groups were assessed using the independent samples t-test. Following the t-test analysis, Pearson's correlation coefficient (r) was used to assess the relationship between lipid parameters and both creatinine-based and cystatin C-based eGFR. This analysis also helped determine which eGFR estimation method showed a stronger correlation with the lipid parameters. Correlations between cystatin C- and creatinine-based eGFR (using CKD-EPI and MDRD equations) and lipid parameters (TC, TG, LDL-C, HDL-C, VLDL-C) in adult CKD patients versus age- and sex-matched healthy controls were analyzed to assess their association with cardiovascular risk in CKD patients.

## Results

A total of 100 individuals were enrolled in the study. The mean age of the CKD group was 46.88±14.39 years (18-70 years). The case group comprised 39 (78%) males and 11 (22%) females. The mean age of the control group was 43.34±12.40 years. The CKD group had markedly elevated serum creatinine and cystatin C levels. TC, TG, LDL-C, VLDL-C, TC/HDL ratio, and LDL/HDL ratio were notably increased. HDL-C and cardioprotective lipids were reduced. Overall, the case group demonstrated higher renal and lipid profiles compared to controls, indicating an adverse lipid profile and increased risk of dyslipidemia among CKD patients (Table 1).

**Table 1:** Comparison of baseline characteristics in the study population Reference range: serum creatinine: 0.7-1.3 mg/dl (male) and 0.6-1.1 mg/dl (female); serum cystatin C: 0.56-1.02 mg/L; TC: <200 mg/dl; TG: <61 mg/dl; HDL-C: 40-60 mg/dl; LDL-C: <100 mg/dl; VLDL-C: <32 mg/dl; TC/HDL ratio: <4; and LDL/HDL ratio: <6 CKD: chronic kidney disease; TC: total cholesterol; TG: triglyceride; LDL-C: low-density lipoprotein cholesterol; HDL-C: high-density lipoprotein cholesterol; VLDL-C: very-low-density lipoprotein cholesterol

Variables	CKD group (n=50)		Control group (n=50)	
	Mean	SD	Mean	SD
Age (years)	46.88	14.39	43.34	12.40
Serum creatinine (mg/dl)	11.64	5.86	0.82	0.17
Serum cystatin C (mg/L)	6.39	2.11	0.63	0.24
TC (mg/dl)	164.22	44.25	150.22	25.28
TG (mg/dl)	157.72	67.34	115.98	19.15
HDL-C (mg/dl)	44.9	4.51	47.4	3.12
LDL-C (mg/dl)	87.78	43.83	79.62	25.02
VLDL-C (mg/dl)	31.54	13.47	23.2	3.83
TC/HDL ratio	3.71	1.14	3.19	0.6
LDL/HDL ratio	1.99	1.07	1.7	0.56

Table 2 showed that the CKD group had significantly elevated serum TG (157.72±67.34 vs. 115.98±19.15 mg/dl; p<0.0001) and VLDL-C (31.54±13.47 vs. 23.2±3.83 mg/dl; p<0.0001) compared to controls. The TC/HDL ratio was significantly higher in the CKD group (3.71±1.14 vs. 3.19±0.6; p=0.0053). HDL-C was significantly reduced in CKD cases (44.90±4.51 vs. 47.4±3.12 mg/dl; p=0.0017). TC (164.22±44.25 vs. 150.22±25.28 mg/dl; p=0.0549), LDL-C (87.78±43.83 vs. 79.62±25.02 mg/dl; p=0.2557), and LDL/HDL ratio (1.99±1.07 vs. 1.7±0.56; p=0.0927) did not differ significantly between groups.

**Table 2:** Comparison of lipid parameters in the study population An independent Student's t-test was used for the means between the two groups. *A p-value of <0.05 is considered statistically significant. CKD: chronic kidney disease; TC: total cholesterol; TG: triglyceride; LDL-C: low-density lipoprotein cholesterol; HDL-C: high-density lipoprotein cholesterol; VLDL-C: very-low-density lipoprotein cholesterol

Parameters	CKD group (mean±SD)	Control group (mean±SD)	P-value	t-value
TC (mg/dl)	164.22±44.25	150.22±25.28	0.0549	-1.943
TG (mg/dl)	157.72±67.34	115.98±19.15	<0.0001*	-4.216
HDL-C (mg/dl)	44.90±4.51	47.4±3.12	0.0017*	3.223
LDL-C (mg/dl)	87.78±43.83	79.62±25.02	0.2557	-1.143
VLDL-C (mg/dl)	31.54±13.47	23.2±3.83	<0.0001*	-4.211
TC/HDL ratio	3.71±1.14	3.19±0.6	0.0053*	-2.854
LDL/HDL ratio	1.99±1.07	1.7±0.56	0.0927	-1.698

As noted in Table 3, none of the lipid parameters (TC, TG, HDL-C, LDL-C, VLDL-C, TC/HDL ratio, LDL/HDL ratio) showed a statistically significant correlation with either cystatin C-based eGFR or creatinine-based eGFR (all p-values above 0.05). LDL-C showed the strongest (though non-significant) negative correlation with cystatin C-based eGFR (r=-0.1913; p=0.183), suggesting that higher LDL-C tends weakly toward lower eGFR by this measure. TC also had a weak negative correlation with cystatin C-based eGFR (r=-0.1603) but none with creatinine-based eGFR. TG and VLDL-C showed identical weak positive correlations (r=+0.1098 for cystatin C-based eGFR; they are mathematically linked since VLDL-C=TG/5). Correlations with creatinine-based eGFR were all extremely weak (|r|<0.08), suggesting lipid parameters had little relationship with creatinine. The discrepancy in direction between cystatin C-based eGFR and creatinine-based eGFR correlations for TC (r=-0.16 vs. r=+0.004) reflects the different sensitivities of the two GFR estimation methods.

**Table 3:** Correlation of lipid parameters with eGFR (cystatin C- and creatinine-based) A p-value of <0.05 is considered statistically significant. r value: Pearson's correlation coefficient (r); NS: non-significant; eGFR: estimated glomerular filtration rate; TC: total cholesterol; TG: triglyceride; LDL-C: low-density lipoprotein cholesterol; HDL-C: high-density lipoprotein cholesterol; VLDL-C: very-low-density lipoprotein cholesterol

Parameter	Cystatin C-based eGFR			Creatinine-based eGFR		
	r	P-value	Significance	r	P-value	Significance
TC	-0.1603	0.2661	NS	0.0039	0.9785	NS
TG	0.1098	0.448	NS	0.0727	0.6161	NS
HDL-C	-0.0418	0.7733	NS	0.0221	0.8788	NS
LDL-C	-0.1913	0.1832	NS	-0.0207	0.8868	NS
VLDL-C	0.1098	0.448	NS	0.0727	0.6161	NS
TC/HDL ratio	-0.1399	0.3325	NS	-0.0097	0.9465	NS
LDL/HDL ratio	-0.1855	0.1971	NS	-0.0327	0.8217	NS

The p-values of cystatin C-based eGFR were generally lower compared to creatinine-based eGFR, especially for LDL-C (p=0.1832), LDL/HDL ratio (p=0.1971), and TC (p=0.2661). This suggests a slightly stronger negative trend between worsening renal function and atherogenic lipid parameters when eGFR was estimated using cystatin C. For eGFR based on creatinine, the p-values were much higher (mostly >0.60), indicating almost no detectable association between lipid parameters and renal function.

The interpretation of correlation coefficients of lipid parameters showed weak negative correlations with cystatin C-based eGFR (TC: r=-0.1603; LDL-C: r=-0.1913; LDL/HDL ratio: r=-0.1855). This means that as renal function declined, lipid parameters tend to increase slightly. In contrast, creatinine-based eGFR correlations were extremely weak and close to zero, suggesting poorer sensitivity in detecting subtle lipid-related renal changes.

## Discussion

The present study evaluated the correlation between cystatin C- and creatinine-based eGFR (using CKD-EPI and MDRD equations) and lipid parameters (TC, TG, LDL-C, HDL-C, VLDL-C) in adult CKD patients versus age- and sex-matched healthy controls. Serum creatinine and cystatin C increased as expected in CKD patients. CKD cases showed significantly higher serum TG and VLDL-C and decreased HDL-C than controls. Cystatin C-based eGFR demonstrated relatively stronger correlations with lipid abnormalities compared to creatinine-based eGFR, although these associations did not reach statistical significance.

DSa et al. found that serum creatinine and cystatin C levels increased significantly in CKD groups (p<0.001) [7]. Our study shows similar results such as higher creatinine and cystatin C as compared to the control group.

Das et al. reported that dyslipidemia was observed in 65% of participants and was more prevalent in advanced CKD stages. A significant decline in HDL-C levels was observed with disease progression. TG levels increased with advancing CKD but were not statistically significant [5]. These findings aligned with our study, where TG, HDL-C, and VLDL-C levels were statistically significant. However, TC, LDL-C, TC/HDL ratio, and LDL/HDL ratio were not statistically significant, which is in contrast with Das et al. Lateef et al. concluded that there was a significant (p≤0.05) decrease in the mean values of HDL-C level in patients with CKD as compared with healthy subjects [8].

Pachpor et al. found that CKD patients had considerably higher mean serum cystatin C levels (3.8±0.96 mg/L) when compared to healthy individuals (0.76±0.11 mg/L), a difference that was statistically significant (p<0.001). This suggests that cystatin C is not just a reliable marker of reduced kidney function but also one that remains unaffected by muscle mass or gender [9]. Consistent with these findings, our study yielded comparable results, with serum cystatin C levels being markedly elevated in CKD patients (6.39±2.11 mg/L) relative to the control group (0.63±0.24 mg/L), further reinforcing the role of cystatin C as a sensitive and reliable indicator of renal dysfunction.

According to Kumar et al., serum cystatin C and LDL-C levels did not differ significantly between patients with CKD and those with both CKD and cardiovascular disease (CVD). However, when examined in CVD patients separately, a clear and significant link between these two markers was observed. This suggests that checking LDL-C levels may not be particularly useful when monitoring CKD patients who are at risk of developing CVD. The authors propose that this is likely because CVD development in CKD patients appears to be driven more by other underlying factors rather than disruptions in cholesterol metabolism [10]. In the general population, higher LDL-C levels are well known to increase cardiovascular risk. However, in patients with advanced kidney disease, this relationship weakens or even reverses, a phenomenon known as "reverse epidemiology". This is believed to occur because the uremic environment itself drives cardiovascular deaths through non-atherosclerotic mechanisms such as heart failure and sudden cardiac death, independent of LDL-C levels [11].

In the present study, cystatin C-based eGFR showed comparatively stronger trends with atherogenic markers such as LDL-C, LDL/HDL ratio, and TC, indicating that as kidney function declines, lipid levels tend to rise gradually. Although these findings did not reach statistical significance, they suggest that cystatin C may be a more sensitive marker than creatinine in capturing early metabolic changes in CKD patients.

Kumar et al. concluded lipid profiles (TC, TG, LDL-C, VLDL-C) except HDL-C to be significantly and negatively correlated with eGFR by serum cystatin C (p<0.05) using the CKD-EPI formula. Even the ratios TC/HDL and LDL/HDL were found to correlate inversely and significantly (p<0.01). Cystatin C-based eGFR showed significant correlation with serum lipids and CVD lipid indices which were not seen with creatinine-based eGFR [12]. In our study, no significant association was found between lipid parameters and either the cystatin C-based or creatinine-based eGFR equations. Although the cystatin C-based eGFR showed a slightly better correlation than the creatinine-based eGFR, the difference was not significant. Overall, our findings suggest that neither eGFR estimation method is suitable for assessing cardiovascular risk based on lipid parameters.

Several limitations of this study need to be considered. The small sample size may have affected the statistical power of our findings. Being a single-center study, the results may not reflect the wider CKD population. No follow-up was carried out to assess the cardiovascular risk over time. The relatively high cost of cystatin C testing may also limit its routine use in clinical practice. As most of our patients were in stage 4 or 5 CKD and disease stage-wise grouping was not performed, future studies including patients across all stages of CKD would provide a better understanding of the relationship between lipid abnormalities and progressive kidney disease. Information on dialysis modality and related treatment details was not collected in this study, which could have given us a better understanding of disease severity and outcomes in patients with stage 4 and 5 CKD. Factors such as dietary habits, statin use, and comorbidities like diabetes and hypertension were not fully accounted for, which may have influenced the overall results.

## Conclusions

CKD patients had an unfavorable lipid profile compared to healthy controls, with significantly higher TG and VLDL-C and lower HDL-C, reflecting the well-known link between impaired kidney function and dyslipidemia. Correlations between eGFR and lipid parameters were weak and did not reach statistical significance for either cystatin C-based or creatinine-based eGFR, although cystatin C-based eGFR showed slightly stronger trends. This suggests that neither eGFR estimate alone may be sufficient to predict lipid-related cardiovascular risk in CKD patients. Larger, multicenter studies with stage-wise CKD classification and different comorbidities should be studied further to better understand the association between eGFR equations and cardiovascular risk in CKD patients.
